# Crystal structures of 3-fluoro-*N*-[2-(tri­fluoro­meth­yl)phen­yl]benzamide, 3-bromo-*N*-[2-(tri­fluoro­meth­yl)phen­yl]benzamide and 3-iodo-*N*-[2-(tri­fluoro­meth­yl)phen­yl]benzamide

**DOI:** 10.1107/S2056989016007866

**Published:** 2016-05-17

**Authors:** P. A. Suchetan, E. Suresha, S. Naveen, N. K. Lokanath

**Affiliations:** a Department of Chemistry, University College of Science, Tumkur University, Tumkur 572 103, India; b Institution of Excellence, University of Mysore, Manasagangotri, Mysuru 570 006, India; c Department of Studies in Physics, University of Mysore, Manasagangotri, Mysuru 570 006, India

**Keywords:** crystal structure, amide, benzamide, 2-(tri­fluoro­meth­yl)phen­yl, N—H⋯O hydrogen bonding, halogen–halogen contacts

## Abstract

In the title compounds, C_14_H_9_F_4_NO, (I), C_14_H_9_BrF_3_NO, (II), and C_14_H_9_F_3_INO, (III), the two benzene rings are inclined to one another by 43.94 (8)° in mol­ecule *A* and 55.66 (7)° in mol­ecule *B* of compound (I), which crystallizes with two independent mol­ecules in the asymmetric unit, but by only 10.40 (12)° in compound (II) and 12.5 (2)° in compound (III). In the crystals of all three compounds, N—H⋯O hydrogen bonds link the mol­ecules to form chains propagating along the *a*-axis direction for (I), and along the *b*-axis direction for (II) and (III). In the crystal of (I), –*A*–*B*–*A*–*B*– chains are linked by C—H⋯O hydrogen bonds, forming layers parallel to (010). Within the layers there are weak offset π–π inter­actions present [inter­centroid distances = 3.868 (1) and 3.855 (1) Å]. In the crystals of (II) and (III), the chains are linked *via* short halogen–halogen contacts [Br⋯Br = 3.6141 (4) Å in (II) and I⋯I = 3.7797 (5) Å in (III)], resulting in the formation of ribbons propagating along the *b*-axis direction.

## Abstract 2

The crystal structures of three *N*-[2-(tri­fluoro­meth­yl)phen­yl]benzamides are reported. The 3-fluoro­benzamide crystallized with two independent mol­ecules in the asymmetric unit; the dihedral angles between the two benzene rings are 43.94 (8) and 55.66 (7)°. In the 3-bromo­benzamide and the 3-iodo­benzamide, this dihedral angle is much smaller, *viz.* 10.40 (12) and 12.5 (2)°, respectively.

## Chemical context   

Amides are very common in nature, and are easily synthesized and provide structural rigidity to various mol­ecules (Gowda *et al.*, 2003 ▸). Furthermore, *N*-aryl­amides show a broad spectrum of pharmacological properties, including anti­bacterial (Manojkumar *et al.*, 2013*a* ▸), anti­tumor (Abdou *et al.*, 2004 ▸), anti­oxidant, analgesic and anti­viral activity (Manojkumar *et al.*, 2013*b* ▸). In view of their importance, the title *N*-(2-tri­fluoro­methyl­phen­yl)benzamides (I)–(III) were synthesized and we report herein on their crystal structures.
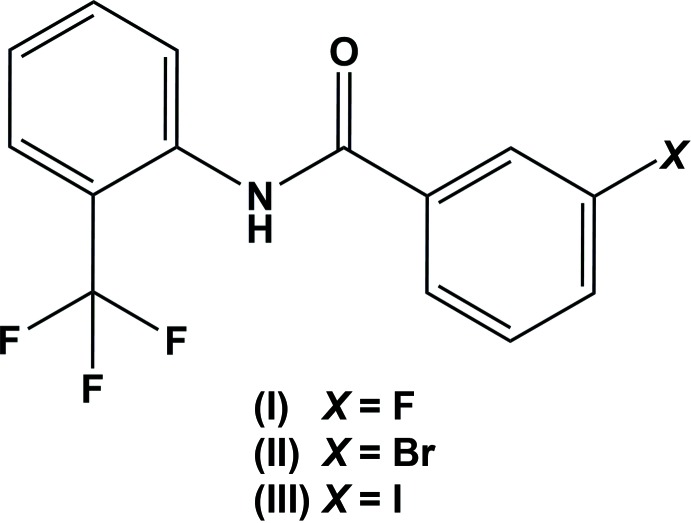


## Structural commentary   

**Figure 1:**
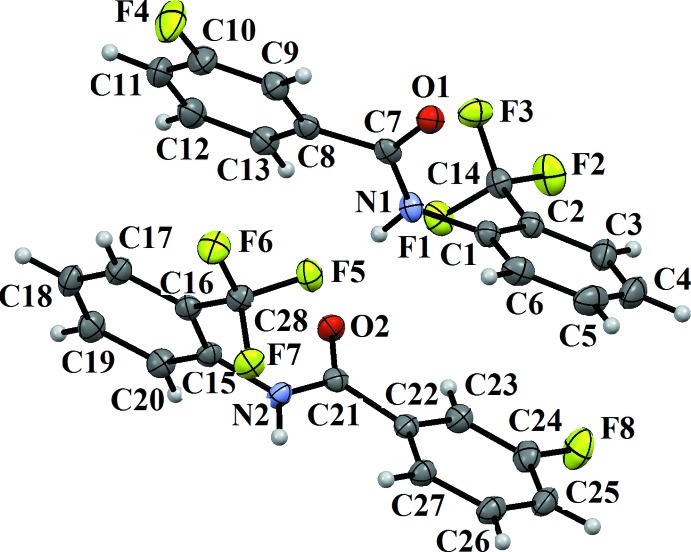
A view of the mol­ecular structure of compound (I), showing the atom labelling. Displacement ellipsoids are drawn at the 50% probability level.

**Figure 2:**
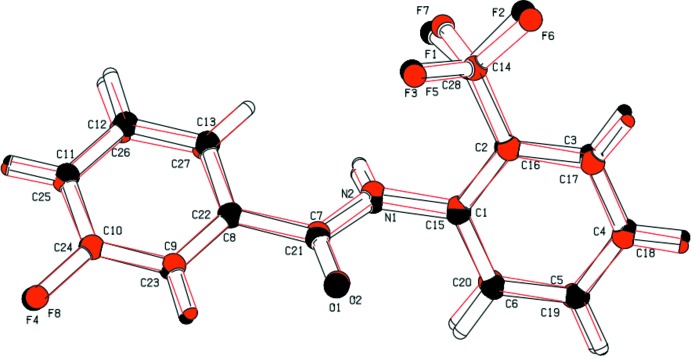
A view of the mol­ecular fit of mol­ecules *A* (black) and *B* (red) of compound (I).

The mol­ecular structure of compound (I) is illustrated in Fig. 1 ▸. It crystallizes with two independent mol­ecules (*A* and *B*) in the asymmetric unit, which slightly differ in their mol­ecular conformations, as shown in the *AutoMolFit* diagram (Fig. 2 ▸; Spek, 2009 ▸). In both mol­ecules, the 3-fluoro substituent on the benzoic acid ring and the 2-CF_3_ substituent on the aniline ring are *anti* to one another, and the 3-fluoro substituent is *anti* to the N—H bond in the central –C_ar_—C(=O)—N—C_ar_– (ar = aromatic) segment of the mol­ecules. The dihedral angle between the two benzene rings is 43.94 (8)° in mol­ecule *A*, while in mol­ecule *B* it is larger, being 55.66 (7)°. The torsion angle of the central –C_ar_—C(=O)—N—C_ar_– segment is 176.74 (12)° in mol­ecule *A* and −179.58 (12)° in mol­ecule *B*.

**Figure 3:**
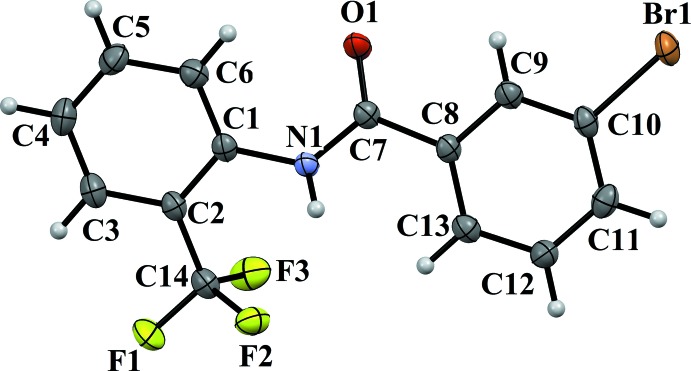
A view of the mol­ecular structure of compound (II), showing the atom labelling. Displacement ellipsoids are drawn at the 50% probability level.

The mol­ecular structures of compounds (II) and (III) are illustrated in Figs. 3 ▸ and 4 ▸, respectively. Here, the 3-bromo and 3-iodo substituents on the benzoic acid ring and the 2-CF_3_ substitution on the aniline ring are *anti* to one another, and the 3-bromo and 3-iodo substituents are *anti* to the N—H bond in the central –C_ar_—C(=O)—N—C_ar_– segment of the mol­ecules, similar to situation observed in (I). The dihedral angle between the two benzene rings is 10.40 (12)° in (II) and 12.5 (2)° in (III), which is much less than observed for mol­ecules *A* and *B* of compound (I). The torsion angle of the central –C_ar_—C(=O)—N—C_ar_– segment is −175.5 (2)° in (II) and 174.8 (3)° in (III), again similar to that in mol­ecules *A* and *B* of compound (I).

## Supra­molecular features   

**Figure 5:**
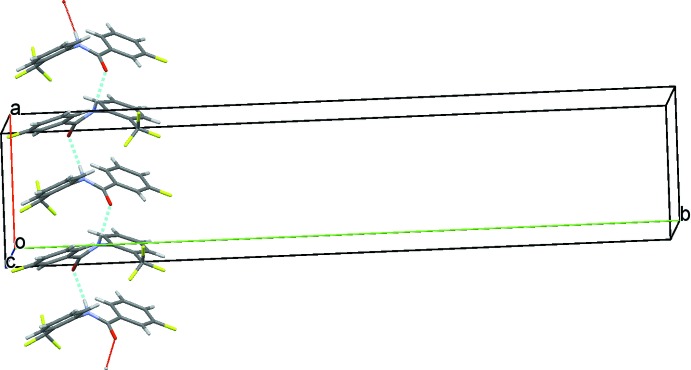
A view along the *c* axis of the crystal packing of compound (I). The N—H⋯O hydrogen bonds are shown as dashed lines (see Table 1 ▸).

**Figure 6:**
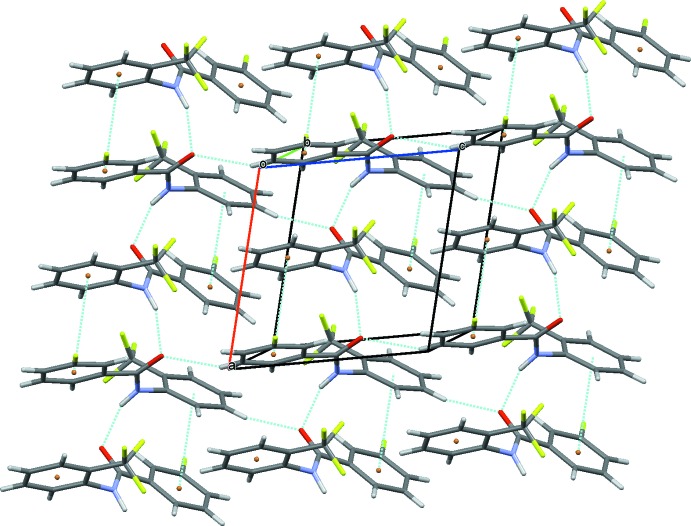
A view along the *b* axis of the crystal packing of compound (I). The C—H⋯O (see Table 1 ▸) and π–π inter­actions are shown as dashed lines.

In the crystal of (I), strong N1—H1⋯O2 and N2—H2⋯O1 hydrogen bonds link the mol­ecules to form –*A*–*B*–*A*–*B*– *C*(4) chains running along the *a-*axis direction (Table 1 ▸ and Fig. 5 ▸). Neighbouring chains are linked *via* C5—H5⋯O2 and C12—H12⋯O1 hydrogen bonds (Table 1 ▸), forming layers lying parallel to the *ac* plane (Fig. 6 ▸). Within the layers there are weak offset π–π inter­actions present involving the aniline and benzoic acid rings [*Cg*1⋯*Cg*4 = 3.8682 (9) Å and *Cg*2⋯*Cg*3^i^ = 3.8553 (9) Å; *Cg*1 and *Cg*3 are the centroids of the aniline rings C1–C6 and C15–C20, respectively; *Cg*2 and *Cg*4 are the centroids of the benzoic acid rings C8–C13 and C22–C27, respectively; symmetry code (i) *x* − 1, *y*, *z*]. The crystal structure does not feature any C—H⋯F or F⋯F inter­actions (Fig. 6 ▸).

**Figure 7:**
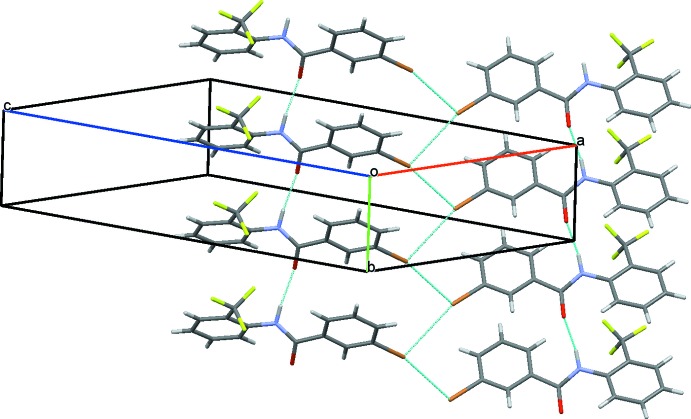
A view along the *b* axis of the crystal packing of compound (II). The N—H⋯O hydrogen bonds (see Table 2 ▸) and the Br⋯Br contacts are shown as dashed lines.

The crystal structure of (II), features strong N1—H1⋯O1 hydrogen bonds (Fig. 7 ▸ and Table 2 ▸) similar to those observed in (I), linking the mol­ecules into *C*(4) chains running parallel to the *b* axis (Fig. 7 ▸). Adjacent chains are connected *via* short Br⋯Br contacts [3.6141 (4) Å], forming ribbons along [010]; see Fig. 7 ▸.

**Figure 8:**
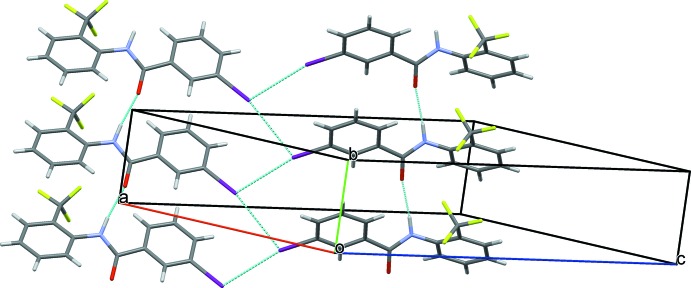
A view along the *b* axis of the crystal packing of compound (III). The N—H⋯O hydrogen bonds (see Table 3 ▸) and the I⋯I contacts are shown as dashed lines.

The crystal structure of (III), features similar characteristics to that of (II). Strong N1—H1⋯O1 hydrogen bonds link the mol­ecules into *C*(4) chains running parallel to the *b* axis (Table 3 ▸ and Fig. 8 ▸). Adjacent chains are linked *via* short I⋯I contacts [3.7797 (5) Å], forming ribbons along [010]; see Fig. 8 ▸.

From the above observations, it can be concluded that the bromo and iodo substitutions on the *meta* position of the benzoic acid ring have a similar effect on the mol­ecular conformations and the supra­molecular architectures exhibited by this class of compounds, whereas the fluoro substitution has a very different influence. For instance, there are two mol­ecules in the asymmetric unit of (I) compared to one mol­ecules in those of (II) and (III). Also, the dihedral angle between the two benzene rings is much larger in the two mol­ecules (*A* and *B*) of (I), compared to the values observed in (II) and (III). Furthermore, the crystal structures of both (II) and (III) feature short halogen⋯halogen contacts, in addition to the N—H⋯O hydrogen bonds, resulting in one-dimensional structures, whereas in (I), in the absence of F⋯F contacts, C—H⋯O hydrogen bonds and π–π inter­actions are observed, in addition to the strong N—H⋯O hydrogen bonds, resulting in a two-dimensional architecture.

## Database survey   

A search of the Cambridge Structural Database (CSD; Version 5.37, update February 2016; Groom *et al.*, 2016 ▸) for similar compounds *viz. N*-(2-(tri­fluoro­meth­yl)phen­yl)aryl­amides, gave four hits. They include *N*-(2-(tri­fluoro­meth­yl)phen­yl)benzamide, for which there are three reports: JOZFUB and JOZFUB01 in space group *P*4_3_ (Hathwar *et al.*, 2014 ▸) and LASHOE in space group *P*4_1_ (Panini & Chopra, 2012 ▸), and 2-(tri­fluoro­meth­yl)-*N*-(2-(tri­fluoro­meth­yl)phen­yl)benzamide (LASKAT; Panini & Chopra, 2012 ▸). In compounds LASHOE and LASKAT, the 2-CF_3_ group in the aniline ring is nearly *syn* to the N—H bond in the central amide segment of the mol­ecule, as observed in the title compounds. In LASHOE (Panini & Chopra, 2012 ▸), the dihedral angle between the two benzene rings is 41.3 (1)°, and the torsion angle of the central –C_ar_—N—C(=O)—C_ar_– segment is 175.1 (5)°, which is very close to the values observed for the two independent mol­ecules in compound (I). This shows that introducing a fluorine atom into the *meta* position of the benzoyl ring, as in compound (I), has little effect on the mol­ecular conformation of this class of compounds.

## Synthesis and crystallization   

The different substituted benzoic acids (3 mmol) were dissolved in phospho­rous oxychloride taken in a 250 ml round-bottomed flask. The mixtures were refluxed for an hour and later cooled to 273 K. An equimolar amount of 2-(tri­fluoro­meth­yl)aniline was added dropwise to these mixtures with continuous stirring. After completion of the addition, the reaction mixtures were brought to room temperature and stirring was continued for 1 h. The reaction mixtures were poured into ice-cold water. The solids that separated were washed thoroughly with water, followed by washing with dilute hydro­chloric acid, water, aqueous sodium hydrogen carbonate solution and again with water. The compounds were filtered under suction, dried and recrystallized from aqueous ethanol to constant melting points. Prismatic colourless single crystals of all three compounds were obtained by slow evaporation of solutions in methanol, with a few drops of water.

## Refinement details   

Crystal data, data collection and structure refinement details are summarized in Table 4 ▸. In all three compounds the NH H atoms were located in difference Fourier maps and refined with a distance restraint: N—H = 0.90 (4) Å. The C-bound H atoms were positioned with idealized geometry and refined using a riding model: C—H = 0.95 Å, with *U*_iso_ = 1.2*U*_eq_(C). In the final cycles of refinement of compound (III), a bad reflection (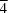 2 2) was omitted, which lead to an improvement in the values of *R*1, *wR*2, and GOF.

## Supplementary Material

Crystal structure: contains datablock(s) I, II, III, global. DOI: 10.1107/S2056989016007866/su5298sup1.cif

Structure factors: contains datablock(s) I. DOI: 10.1107/S2056989016007866/su5298Isup2.hkl

Structure factors: contains datablock(s) II. DOI: 10.1107/S2056989016007866/su5298IIsup3.hkl

Structure factors: contains datablock(s) III. DOI: 10.1107/S2056989016007866/su5298IIIsup4.hkl

Supporting information file. DOI: 10.1107/S2056989016007866/su5298Isup5.cml

Supporting information file. DOI: 10.1107/S2056989016007866/su5298IIsup6.cml

Supporting information file. DOI: 10.1107/S2056989016007866/su5298IIIsup7.cml

CCDC references: 1479657, 1479656, 1479655

Additional supporting information:  crystallographic information; 3D view; checkCIF report

## Figures and Tables

**Figure 4:**
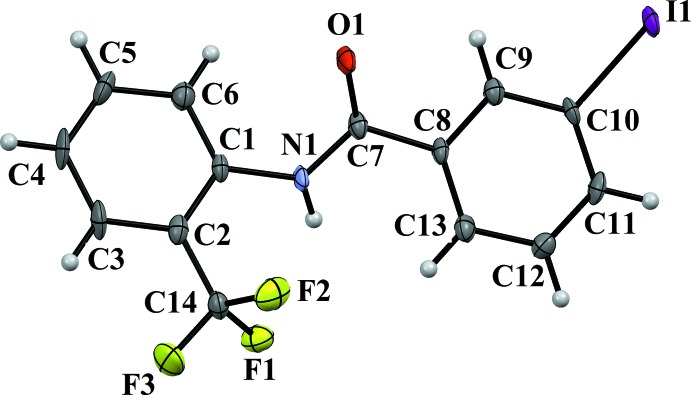
A view of the mol­ecular structure of compound (III), showing the atom labelling. Displacement ellipsoids are drawn at the 50% probability level.

**Table 1:** Hydrogen-bond geometry (Å, °) for (I)

*D*—H⋯*A*	*D*—H	H⋯*A*	*D*⋯*A*	*D*—H⋯*A*
N1—H1⋯O2	0.87 (2)	2.01 (2)	2.8239 (16)	157 (1)
N2—H2⋯O1^i^	0.89 (2)	1.99 (2)	2.8303 (16)	158 (1)
C5—H5⋯O2^ii^	0.95	2.35	3.2861 (18)	167
C12—H12⋯O1^iii^	0.95	2.45	3.3172 (17)	152

Symmetry codes: (i) 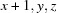; (ii) 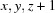; (iii) 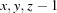.

**Table 2:** Hydrogen-bond geometry (Å, °) for (II)

*D*—H⋯*A*	*D*—H	H⋯*A*	*D*⋯*A*	*D*—H⋯*A*
N1—H1⋯O1^i^	0.89 (2)	2.00 (2)	2.835 (2)	156 (3)

Symmetry code: (i) 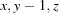.

**Table 3:** Hydrogen-bond geometry (Å, °) for (III)

*D*—H⋯*A*	*D*—H	H⋯*A*	*D*⋯*A*	*D*—H⋯*A*
N1—H1⋯O1^i^	0.89 (3)	1.99 (4)	2.826 (5)	156 (5)

Symmetry code: (i) 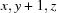.

**Table 4:** Experimental details

	(I)	(II)	(III)
Crystal data			
Chemical formula	C_14_H_9_F_4_NO	C_14_H_9_BrF_3_NO	C_14_H_9_F_3_INO
*M*_r_	283.22	344.13	391.12
Crystal system, space group	Monoclinic, *P*2_1_/*c*	Monoclinic, *P*2_1_/*n*	Monoclinic, *P*2_1_/*n*
Temperature (K)	173	173	173
*a*, *b*, *c* (Å)	8.0258 (2), 39.7598 (12), 7.8932 (2)	12.9456 (6), 4.7377 (2), 21.9025 (10)	13.3358 (6), 4.7471 (2), 22.3558 (10)
β (°)	103.937 (1)	104.770 (2)	105.848 (2)
*V* (Å^3^)	2444.60 (11)	1298.94 (10)	1361.47 (10)
*Z*	8	4	4
Radiation type	Cu *K*α	Cu *K*α	Cu *K*α
μ (mm^−1^)	1.22	4.63	18.78
Crystal size (mm)	0.29 × 0.22 × 0.19	0.28 × 0.24 × 0.20	0.27 × 0.22 × 0.18

Data collection			
Diffractometer	Bruker APEXII CCD	Bruker APEXII CCD	Bruker APEXII CCD
Absorption correction	Multi-scan (*SADABS*; Bruker, 2009 ▸)	Multi-scan (*SADABS*; Bruker, 2009 ▸)	Multi-scan (*SADABS*; Bruker, 2009 ▸)
*T*_min_, *T*_max_	0.760, 0.793	0.315, 0.396	0.081, 0.133
No. of measured, independent and observed [*I* > 2σ(*I*)] reflections	13874, 3997, 3816	8466, 2114, 1986	7120, 2223, 2124
*R*_int_	0.034	0.039	0.053
(sin θ/λ)_max_ (Å^−1^)	0.584	0.585	0.584

Refinement			
*R*[*F* ^2^ > 2σ(*F* ^2^)], *wR*(*F* ^2^), *S*	0.033, 0.091, 1.06	0.034, 0.090, 1.05	0.043, 0.109, 1.09
No. of reflections	3997	2114	2223
No. of parameters	369	185	185
No. of restraints	2	1	1
H-atom treatment	H atoms treated by a mixture of independent and constrained refinement	H atoms treated by a mixture of independent and constrained refinement	H atoms treated by a mixture of independent and constrained refinement
Δρ_max_, Δρ_min_ (e Å^−3^)	0.19, −0.17	0.62, −0.34	1.84, −1.41

Computer programs: *APEX2*, *SAINT-Plus* and *XPREP* (Bruker, 2009 ▸), *SHELXS97* and *SHELXL97* (Sheldrick, 2008 ▸) and *Mercury* (Macrae *et al.*, 2008 ▸).

## supplementary crystallographic information

### (I) 3-Fluoro-N-[2-(trifluoromethyl)phenyl]benzamide Crystal data

**Table:**

C_14_H_9_F_4_NO	Prism
*M**_r_* = 283.22	*D*_x_ = 1.539 Mg m^−^^3^
Monoclinic, *P*2_1_/*c*	Melting point: 377 K
*a* = 8.0258 (2) Å	Cu *K*α radiation, λ = 1.54178 Å
*b* = 39.7598 (12) Å	Cell parameters from 143 reflections
*c* = 7.8932 (2) Å	θ = 2.2–64.2°
β = 103.937 (1)°	µ = 1.22 mm^−^^1^
*V* = 2444.60 (11) Å^3^	*T* = 173 K
*Z* = 8	Prism, colourless
*F*(000) = 1152	0.29 × 0.22 × 0.19 mm

### (I) 3-Fluoro-N-[2-(trifluoromethyl)phenyl]benzamide Data collection

**Table:**

Bruker APEXII CCD diffractometer	3997 independent reflections
Radiation source: fine-focus sealed tube	3816 reflections with *I* > 2σ(*I*)
Graphite monochromator	*R*_int_ = 0.034
phi and φ scans	θ_max_ = 64.2°, θ_min_ = 2.2°
Absorption correction: multi-scan (SADABS; Bruker, 2009)	*h* = −9→7
*T*_min_ = 0.760, *T*_max_ = 0.793	*k* = −44→45
13874 measured reflections	*l* = −6→9

### (I) 3-Fluoro-N-[2-(trifluoromethyl)phenyl]benzamide Refinement

**Table:**

Refinement on *F*^2^	Primary atom site location: structure-invariant direct methods
Least-squares matrix: full	Secondary atom site location: difference Fourier map
*R*[*F*^2^ > 2σ(*F*^2^)] = 0.033	Hydrogen site location: inferred from neighbouring sites
*wR*(*F*^2^) = 0.091	H atoms treated by a mixture of independent and constrained refinement
*S* = 1.06	*w* = 1/[σ^2^(*F*_o_^2^) + (0.0464*P*)^2^ + 0.8967*P*] where *P* = (*F*_o_^2^ + 2*F*_c_^2^)/3
3997 reflections	(Δ/σ)_max_ < 0.001
369 parameters	Δρ_max_ = 0.19 e Å^−^^3^
2 restraints	Δρ_min_ = −0.17 e Å^−^^3^

### (I) 3-Fluoro-N-[2-(trifluoromethyl)phenyl]benzamide Special details

**Table:**

Geometry. All s.u.'s (except the s.u. in the dihedral angle between two l.s. planes) are estimated using the full covariance matrix. The cell s.u.'s are taken into account individually in the estimation of s.u.'s in distances, angles and torsion angles; correlations between s.u.'s in cell parameters are only used when they are defined by crystal symmetry. An approximate (isotropic) treatment of cell s.u.'s is used for estimating s.u.'s involving l.s. planes.
Refinement. Refinement of F^2^ against ALL reflections. The weighted R-factor wR and goodness of fit S are based on F^2^, conventional R-factors R are based on F, with F set to zero for negative F^2^. The threshold expression of F^2^ > 2σ(F^2^) is used only for calculating R-factors(gt) etc. and is not relevant to the choice of reflections for refinement. R-factors based on F^2^ are statistically about twice as large as those based on F, and R- factors based on ALL data will be even larger.

### (I) 3-Fluoro-N-[2-(trifluoromethyl)phenyl]benzamide Fractional atomic coordinates and isotropic or equivalent isotropic displacement parameters (Å^2^)

**Table:**

	*x*	*y*	*z*	*U*_iso_*/*U*_eq_	
H2	0.667 (2)	0.1103 (4)	0.558 (2)	0.034 (5)*	
H1	0.140 (2)	0.1376 (4)	0.456 (2)	0.029 (4)*	
F5	0.33100 (11)	0.07085 (2)	0.60352 (10)	0.0292 (2)	
F6	0.35108 (12)	0.01935 (2)	0.52831 (11)	0.0329 (2)	
F7	0.57809 (11)	0.04694 (2)	0.65368 (10)	0.0292 (2)	
O2	0.35341 (12)	0.15177 (2)	0.38529 (12)	0.0247 (2)	
N2	0.56532 (16)	0.11452 (3)	0.48712 (15)	0.0222 (3)	
F8	0.45852 (14)	0.24815 (2)	0.79593 (13)	0.0436 (3)	
C21	0.48174 (17)	0.14343 (3)	0.49780 (17)	0.0198 (3)	
C22	0.54636 (17)	0.16483 (3)	0.65650 (17)	0.0210 (3)	
C28	0.42663 (18)	0.04946 (3)	0.53435 (18)	0.0234 (3)	
C15	0.51404 (17)	0.09248 (3)	0.34014 (17)	0.0209 (3)	
C20	0.53464 (19)	0.10260 (4)	0.17839 (19)	0.0254 (3)	
H20	0.5775	0.1244	0.1644	0.030*	
C23	0.47773 (19)	0.19705 (4)	0.65248 (18)	0.0252 (3)	
H23	0.3996	0.2052	0.5506	0.030*	
C27	0.66387 (18)	0.15357 (4)	0.80616 (18)	0.0238 (3)	
H27	0.7132	0.1318	0.8083	0.029*	
C24	0.5257 (2)	0.21680 (4)	0.7997 (2)	0.0291 (3)	
C26	0.70845 (19)	0.17425 (4)	0.95187 (19)	0.0280 (3)	
H26	0.7879	0.1664	1.0537	0.034*	
C25	0.6385 (2)	0.20607 (4)	0.95048 (19)	0.0291 (3)	
H25	0.6675	0.2201	1.0506	0.035*	
C19	0.49280 (19)	0.08093 (4)	0.03697 (18)	0.0280 (3)	
H19	0.5084	0.0878	−0.0734	0.034*	
C18	0.4285 (2)	0.04928 (4)	0.05599 (19)	0.0283 (3)	
H18	0.3997	0.0345	−0.0413	0.034*	
C17	0.40585 (19)	0.03907 (4)	0.21705 (18)	0.0254 (3)	
H17	0.3613	0.0173	0.2300	0.030*	
C16	0.44841 (17)	0.06066 (4)	0.35957 (18)	0.0212 (3)	
F1	0.01785 (11)	0.19821 (2)	0.34114 (10)	0.0316 (2)	
F3	−0.21044 (11)	0.17739 (2)	0.39516 (11)	0.0316 (2)	
F2	−0.14591 (13)	0.22917 (2)	0.45373 (12)	0.0392 (2)	
O1	−0.08868 (12)	0.09581 (2)	0.62751 (12)	0.0247 (2)	
F4	−0.13792 (16)	−0.00092 (3)	0.21276 (14)	0.0509 (3)	
N1	0.07498 (16)	0.13350 (3)	0.52692 (15)	0.0227 (3)	
C7	−0.01510 (17)	0.10475 (3)	0.51462 (17)	0.0211 (3)	
C13	−0.00037 (19)	0.09805 (4)	0.19810 (19)	0.0263 (3)	
H13	0.0340	0.1209	0.1952	0.032*	
C8	−0.02734 (17)	0.08437 (4)	0.35245 (18)	0.0223 (3)	
C14	−0.07845 (19)	0.19824 (4)	0.45914 (18)	0.0243 (3)	
C1	0.09082 (18)	0.15641 (4)	0.66916 (18)	0.0221 (3)	
C2	0.02149 (18)	0.18867 (4)	0.63846 (18)	0.0223 (3)	
C6	0.17972 (19)	0.14752 (4)	0.83585 (19)	0.0291 (3)	
H6	0.2254	0.1255	0.8577	0.035*	
C9	−0.07529 (19)	0.05080 (4)	0.3572 (2)	0.0275 (3)	
H9	−0.0945	0.0411	0.4610	0.033*	
C12	−0.0237 (2)	0.07837 (4)	0.04937 (19)	0.0310 (3)	
H12	−0.0068	0.0879	−0.0557	0.037*	
C3	0.0453 (2)	0.21184 (4)	0.7738 (2)	0.0300 (3)	
H3	−0.0009	0.2339	0.7528	0.036*	
C10	−0.0941 (2)	0.03200 (4)	0.2076 (2)	0.0325 (4)	
C11	−0.0716 (2)	0.04501 (4)	0.0527 (2)	0.0334 (4)	
H11	−0.0886	0.0314	−0.0490	0.040*	
C5	0.2025 (2)	0.17060 (5)	0.9713 (2)	0.0357 (4)	
H5	0.2633	0.1643	1.0856	0.043*	
C4	0.1367 (2)	0.20270 (4)	0.9394 (2)	0.0364 (4)	
H4	0.1543	0.2186	1.0318	0.044*	

### (I) 3-Fluoro-N-[2-(trifluoromethyl)phenyl]benzamide Atomic displacement parameters (Å^2^)

**Table:**

	*U*^11^	*U*^22^	*U*^33^	*U*^12^	*U*^13^	*U*^23^
F5	0.0319 (5)	0.0342 (5)	0.0230 (4)	0.0058 (4)	0.0097 (3)	−0.0001 (3)
F6	0.0415 (5)	0.0273 (5)	0.0320 (5)	−0.0069 (4)	0.0132 (4)	0.0028 (4)
F7	0.0296 (5)	0.0343 (5)	0.0208 (4)	0.0041 (4)	0.0002 (3)	0.0055 (3)
O2	0.0234 (5)	0.0309 (6)	0.0180 (5)	0.0025 (4)	0.0015 (4)	0.0013 (4)
N2	0.0225 (6)	0.0228 (6)	0.0182 (6)	−0.0006 (5)	−0.0009 (5)	−0.0026 (5)
F8	0.0592 (7)	0.0246 (5)	0.0447 (6)	0.0047 (4)	0.0078 (5)	−0.0076 (4)
C21	0.0200 (7)	0.0234 (7)	0.0170 (6)	−0.0028 (5)	0.0061 (5)	0.0024 (5)
C22	0.0216 (7)	0.0234 (7)	0.0190 (7)	−0.0043 (5)	0.0070 (5)	0.0002 (5)
C28	0.0243 (7)	0.0229 (7)	0.0223 (7)	0.0008 (6)	0.0042 (6)	0.0006 (5)
C15	0.0197 (7)	0.0230 (7)	0.0182 (7)	0.0010 (5)	0.0011 (5)	−0.0014 (5)
C20	0.0272 (8)	0.0250 (7)	0.0236 (7)	−0.0005 (6)	0.0055 (6)	0.0022 (6)
C23	0.0275 (7)	0.0247 (7)	0.0230 (7)	−0.0017 (6)	0.0057 (6)	0.0013 (6)
C27	0.0246 (7)	0.0258 (7)	0.0204 (7)	−0.0018 (6)	0.0041 (6)	0.0001 (6)
C24	0.0357 (8)	0.0209 (7)	0.0328 (8)	−0.0026 (6)	0.0124 (7)	−0.0031 (6)
C26	0.0298 (8)	0.0331 (8)	0.0196 (7)	−0.0053 (6)	0.0032 (6)	−0.0007 (6)
C25	0.0345 (8)	0.0308 (8)	0.0227 (7)	−0.0105 (6)	0.0081 (6)	−0.0072 (6)
C19	0.0309 (8)	0.0346 (8)	0.0180 (7)	0.0021 (6)	0.0053 (6)	0.0020 (6)
C18	0.0327 (8)	0.0294 (8)	0.0210 (7)	0.0032 (6)	0.0029 (6)	−0.0055 (6)
C17	0.0267 (8)	0.0238 (7)	0.0241 (7)	0.0002 (6)	0.0030 (6)	−0.0027 (6)
C16	0.0199 (7)	0.0235 (7)	0.0193 (7)	0.0023 (5)	0.0028 (5)	0.0008 (6)
F1	0.0357 (5)	0.0376 (5)	0.0238 (4)	0.0038 (4)	0.0115 (4)	0.0090 (4)
F3	0.0284 (5)	0.0391 (5)	0.0241 (4)	−0.0029 (4)	0.0002 (3)	0.0043 (4)
F2	0.0486 (6)	0.0281 (5)	0.0412 (5)	0.0161 (4)	0.0112 (4)	0.0085 (4)
O1	0.0253 (5)	0.0299 (5)	0.0187 (5)	−0.0009 (4)	0.0051 (4)	0.0028 (4)
F4	0.0739 (8)	0.0355 (6)	0.0510 (6)	−0.0214 (5)	0.0300 (6)	−0.0176 (5)
N1	0.0272 (6)	0.0217 (6)	0.0213 (6)	0.0022 (5)	0.0096 (5)	0.0012 (5)
C7	0.0187 (7)	0.0241 (7)	0.0194 (7)	0.0062 (5)	0.0025 (5)	0.0046 (5)
C13	0.0262 (7)	0.0295 (8)	0.0230 (7)	0.0023 (6)	0.0054 (6)	0.0037 (6)
C8	0.0186 (7)	0.0265 (7)	0.0213 (7)	0.0027 (5)	0.0038 (5)	0.0014 (6)
C14	0.0271 (7)	0.0221 (7)	0.0246 (7)	0.0033 (6)	0.0081 (6)	0.0025 (6)
C1	0.0219 (7)	0.0251 (7)	0.0197 (7)	−0.0002 (6)	0.0061 (5)	0.0009 (5)
C2	0.0234 (7)	0.0232 (7)	0.0214 (7)	−0.0005 (6)	0.0072 (5)	0.0005 (6)
C6	0.0267 (8)	0.0348 (8)	0.0247 (8)	0.0019 (6)	0.0040 (6)	0.0072 (6)
C9	0.0284 (8)	0.0295 (8)	0.0258 (7)	−0.0019 (6)	0.0088 (6)	−0.0003 (6)
C12	0.0288 (8)	0.0437 (9)	0.0203 (7)	0.0016 (7)	0.0057 (6)	0.0014 (6)
C3	0.0359 (9)	0.0274 (8)	0.0287 (8)	−0.0034 (6)	0.0115 (7)	−0.0060 (6)
C10	0.0325 (8)	0.0307 (8)	0.0357 (9)	−0.0069 (7)	0.0112 (7)	−0.0077 (7)
C11	0.0298 (8)	0.0444 (10)	0.0264 (8)	−0.0021 (7)	0.0074 (6)	−0.0112 (7)
C5	0.0318 (8)	0.0542 (11)	0.0191 (7)	−0.0077 (8)	0.0022 (6)	0.0032 (7)
C4	0.0410 (9)	0.0450 (10)	0.0244 (8)	−0.0117 (8)	0.0100 (7)	−0.0115 (7)

### (I) 3-Fluoro-N-[2-(trifluoromethyl)phenyl]benzamide Geometric parameters (Å, º)

**Table:**

F5—C28	1.3462 (16)	F1—C14	1.3459 (17)
F6—C28	1.3377 (16)	F3—C14	1.3440 (17)
F7—C28	1.3503 (17)	F2—C14	1.3403 (17)
O2—C21	1.2324 (17)	O1—C7	1.2338 (17)
N2—H2	0.889 (18)	F4—C10	1.3583 (19)
N2—C21	1.3438 (18)	N1—H1	0.868 (18)
N2—C15	1.4327 (18)	N1—C7	1.3437 (19)
F8—C24	1.3557 (18)	N1—C1	1.4273 (18)
C21—C22	1.4998 (19)	C7—C8	1.498 (2)
C22—C23	1.392 (2)	C13—H13	0.9500
C22—C27	1.396 (2)	C13—C8	1.398 (2)
C28—C16	1.4993 (19)	C13—C12	1.385 (2)
C15—C20	1.386 (2)	C8—C9	1.392 (2)
C15—C16	1.393 (2)	C14—C2	1.498 (2)
C20—H20	0.9500	C1—C2	1.396 (2)
C20—C19	1.386 (2)	C1—C6	1.383 (2)
C23—H23	0.9500	C2—C3	1.388 (2)
C23—C24	1.378 (2)	C6—H6	0.9500
C27—H27	0.9500	C6—C5	1.387 (2)
C27—C26	1.389 (2)	C9—H9	0.9500
C24—C25	1.378 (2)	C9—C10	1.375 (2)
C26—H26	0.9500	C12—H12	0.9500
C26—C25	1.383 (2)	C12—C11	1.383 (2)
C25—H25	0.9500	C3—H3	0.9500
C19—H19	0.9500	C3—C4	1.385 (2)
C19—C18	1.382 (2)	C10—C11	1.378 (2)
C18—H18	0.9500	C11—H11	0.9500
C18—C17	1.387 (2)	C5—H5	0.9500
C17—H17	0.9500	C5—C4	1.381 (3)
C17—C16	1.391 (2)	C4—H4	0.9500
			
C21—N2—H2	121.3 (12)	C7—N1—H1	120.6 (11)
C21—N2—C15	121.57 (12)	C7—N1—C1	122.97 (12)
C15—N2—H2	115.8 (12)	C1—N1—H1	115.8 (11)
O2—C21—N2	121.88 (12)	O1—C7—N1	122.38 (13)
O2—C21—C22	120.67 (12)	O1—C7—C8	121.14 (13)
N2—C21—C22	117.41 (12)	N1—C7—C8	116.45 (12)
C23—C22—C21	116.60 (12)	C8—C13—H13	120.0
C23—C22—C27	119.84 (13)	C12—C13—H13	120.0
C27—C22—C21	123.51 (13)	C12—C13—C8	120.07 (14)
F5—C28—F7	105.63 (11)	C13—C8—C7	122.83 (13)
F5—C28—C16	112.95 (11)	C9—C8—C7	117.21 (12)
F6—C28—F5	106.38 (11)	C9—C8—C13	119.90 (13)
F6—C28—F7	106.41 (11)	F1—C14—C2	112.80 (12)
F6—C28—C16	112.65 (11)	F3—C14—F1	105.76 (11)
F7—C28—C16	112.27 (11)	F3—C14—C2	113.12 (11)
C20—C15—N2	119.58 (12)	F2—C14—F1	105.87 (11)
C20—C15—C16	119.81 (13)	F2—C14—F3	106.14 (11)
C16—C15—N2	120.58 (12)	F2—C14—C2	112.54 (12)
C15—C20—H20	119.9	C2—C1—N1	119.57 (12)
C19—C20—C15	120.13 (13)	C6—C1—N1	120.87 (13)
C19—C20—H20	119.9	C6—C1—C2	119.52 (13)
C22—C23—H23	120.8	C1—C2—C14	119.84 (12)
C24—C23—C22	118.46 (13)	C3—C2—C14	120.09 (13)
C24—C23—H23	120.8	C3—C2—C1	120.06 (13)
C22—C27—H27	120.1	C1—C6—H6	119.8
C26—C27—C22	119.85 (14)	C1—C6—C5	120.40 (15)
C26—C27—H27	120.1	C5—C6—H6	119.8
F8—C24—C23	118.46 (14)	C8—C9—H9	120.9
F8—C24—C25	118.60 (13)	C10—C9—C8	118.16 (14)
C25—C24—C23	122.93 (14)	C10—C9—H9	120.9
C27—C26—H26	119.6	C13—C12—H12	119.8
C25—C26—C27	120.80 (14)	C11—C12—C13	120.45 (14)
C25—C26—H26	119.6	C11—C12—H12	119.8
C24—C25—C26	118.10 (13)	C2—C3—H3	120.1
C24—C25—H25	121.0	C4—C3—C2	119.71 (15)
C26—C25—H25	121.0	C4—C3—H3	120.1
C20—C19—H19	119.9	F4—C10—C9	118.27 (14)
C18—C19—C20	120.20 (13)	F4—C10—C11	118.59 (14)
C18—C19—H19	119.9	C9—C10—C11	123.14 (15)
C19—C18—H18	120.0	C12—C11—H11	120.9
C19—C18—C17	120.04 (13)	C10—C11—C12	118.26 (14)
C17—C18—H18	120.0	C10—C11—H11	120.9
C18—C17—H17	120.0	C6—C5—H5	120.1
C18—C17—C16	119.99 (14)	C4—C5—C6	119.84 (14)
C16—C17—H17	120.0	C4—C5—H5	120.1
C15—C16—C28	120.12 (12)	C3—C4—H4	119.8
C17—C16—C28	120.05 (13)	C5—C4—C3	120.45 (14)
C17—C16—C15	119.82 (13)	C5—C4—H4	119.8
			
F5—C28—C16—C15	54.98 (17)	F1—C14—C2—C1	−64.46 (17)
F5—C28—C16—C17	−126.11 (14)	F1—C14—C2—C3	115.45 (14)
F6—C28—C16—C15	175.54 (12)	F3—C14—C2—C1	55.52 (17)
F6—C28—C16—C17	−5.54 (18)	F3—C14—C2—C3	−124.56 (14)
F7—C28—C16—C15	−64.33 (16)	F2—C14—C2—C1	175.83 (12)
F7—C28—C16—C17	114.58 (14)	F2—C14—C2—C3	−4.26 (19)
O2—C21—C22—C23	−12.78 (18)	O1—C7—C8—C13	157.12 (13)
O2—C21—C22—C27	164.63 (13)	O1—C7—C8—C9	−19.98 (19)
N2—C21—C22—C23	169.55 (12)	F4—C10—C11—C12	−178.75 (14)
N2—C21—C22—C27	−13.04 (19)	N1—C7—C8—C13	−20.83 (19)
N2—C15—C20—C19	177.07 (13)	N1—C7—C8—C9	162.08 (13)
N2—C15—C16—C28	1.5 (2)	N1—C1—C2—C14	3.9 (2)
N2—C15—C16—C17	−177.37 (13)	N1—C1—C2—C3	−176.01 (13)
F8—C24—C25—C26	−178.96 (13)	N1—C1—C6—C5	176.39 (14)
C21—N2—C15—C20	68.06 (18)	C7—N1—C1—C2	−115.64 (15)
C21—N2—C15—C16	−113.80 (15)	C7—N1—C1—C6	66.89 (19)
C21—C22—C23—C24	176.07 (13)	C7—C8—C9—C10	177.40 (13)
C21—C22—C27—C26	−175.71 (13)	C13—C8—C9—C10	0.2 (2)
C22—C23—C24—F8	−179.84 (13)	C13—C12—C11—C10	−0.4 (2)
C22—C23—C24—C25	0.0 (2)	C8—C13—C12—C11	−0.8 (2)
C22—C27—C26—C25	−0.4 (2)	C8—C9—C10—F4	178.85 (14)
C15—N2—C21—O2	2.8 (2)	C8—C9—C10—C11	−1.5 (2)
C15—N2—C21—C22	−179.58 (12)	C14—C2—C3—C4	179.50 (14)
C15—C20—C19—C18	0.8 (2)	C1—N1—C7—O1	−1.2 (2)
C20—C15—C16—C28	179.68 (13)	C1—N1—C7—C8	176.74 (12)
C20—C15—C16—C17	0.8 (2)	C1—C2—C3—C4	−0.6 (2)
C20—C19—C18—C17	−0.1 (2)	C1—C6—C5—C4	−0.3 (2)
C23—C22—C27—C26	1.6 (2)	C2—C1—C6—C5	−1.1 (2)
C23—C24—C25—C26	1.2 (2)	C2—C3—C4—C5	−0.8 (2)
C27—C22—C23—C24	−1.4 (2)	C6—C1—C2—C14	−178.59 (13)
C27—C26—C25—C24	−1.0 (2)	C6—C1—C2—C3	1.5 (2)
C19—C18—C17—C16	−0.2 (2)	C6—C5—C4—C3	1.2 (2)
C18—C17—C16—C28	−179.05 (13)	C9—C10—C11—C12	1.6 (3)
C18—C17—C16—C15	−0.1 (2)	C12—C13—C8—C7	−176.08 (13)
C16—C15—C20—C19	−1.1 (2)	C12—C13—C8—C9	0.9 (2)

### (I) 3-Fluoro-N-[2-(trifluoromethyl)phenyl]benzamide Hydrogen-bond geometry (Å, º)

**Table:**

*D*—H···*A*	*D*—H	H···*A*	*D*···*A*	*D*—H···*A*
N1—H1···O2	0.87 (2)	2.01 (2)	2.8239 (16)	157 (1)
N2—H2···O1^i^	0.89 (2)	1.99 (2)	2.8303 (16)	158 (1)
C5—H5···O2^ii^	0.95	2.35	3.2861 (18)	167
C12—H12···O1^iii^	0.95	2.45	3.3172 (17)	152

Symmetry codes: (i) *x*+1, *y*, *z*; (ii) *x*, *y*, *z*+1; (iii) *x*, *y*, *z*−1.

### (II) 3-Bromo-N-[2-(trifluoromethyl)phenyl]benzamide Crystal data

**Table:**

C_14_H_9_BrF_3_NO	Prism
*M**_r_* = 344.13	*D*_x_ = 1.760 Mg m^−^^3^
Monoclinic, *P*2_1_/*n*	Melting point: 369 K
*a* = 12.9456 (6) Å	Cu *K*α radiation, λ = 1.54178 Å
*b* = 4.7377 (2) Å	Cell parameters from 132 reflections
*c* = 21.9025 (10) Å	θ = 6.4–64.4°
β = 104.770 (2)°	µ = 4.63 mm^−^^1^
*V* = 1298.94 (10) Å^3^	*T* = 173 K
*Z* = 4	Prism, colourless
*F*(000) = 680	0.28 × 0.24 × 0.20 mm

### (II) 3-Bromo-N-[2-(trifluoromethyl)phenyl]benzamide Data collection

**Table:**

Bruker APEXII CCD diffractometer	2114 independent reflections
Radiation source: fine-focus sealed tube	1986 reflections with *I* > 2σ(*I*)
Graphite monochromator	*R*_int_ = 0.039
phi and φ scans	θ_max_ = 64.4°, θ_min_ = 6.4°
Absorption correction: multi-scan (SADABS; Bruker, 2009)	*h* = −14→15
*T*_min_ = 0.315, *T*_max_ = 0.396	*k* = −5→4
8466 measured reflections	*l* = −24→25

### (II) 3-Bromo-N-[2-(trifluoromethyl)phenyl]benzamide Refinement

**Table:**

Refinement on *F*^2^	Primary atom site location: structure-invariant direct methods
Least-squares matrix: full	Secondary atom site location: difference Fourier map
*R*[*F*^2^ > 2σ(*F*^2^)] = 0.034	Hydrogen site location: inferred from neighbouring sites
*wR*(*F*^2^) = 0.090	H atoms treated by a mixture of independent and constrained refinement
*S* = 1.05	*w* = 1/[σ^2^(*F*_o_^2^) + (0.0627*P*)^2^ + 0.3564*P*] where *P* = (*F*_o_^2^ + 2*F*_c_^2^)/3
2114 reflections	(Δ/σ)_max_ = 0.001
185 parameters	Δρ_max_ = 0.62 e Å^−^^3^
1 restraint	Δρ_min_ = −0.34 e Å^−^^3^

### (II) 3-Bromo-N-[2-(trifluoromethyl)phenyl]benzamide Special details

**Table:**

Geometry. All s.u.'s (except the s.u. in the dihedral angle between two l.s. planes) are estimated using the full covariance matrix. The cell s.u.'s are taken into account individually in the estimation of s.u.'s in distances, angles and torsion angles; correlations between s.u.'s in cell parameters are only used when they are defined by crystal symmetry. An approximate (isotropic) treatment of cell s.u.'s is used for estimating s.u.'s involving l.s. planes.
Refinement. Refinement of F^2^ against ALL reflections. The weighted R-factor wR and goodness of fit S are based on F^2^, conventional R-factors R are based on F, with F set to zero for negative F^2^. The threshold expression of F^2^ > 2σ(F^2^) is used only for calculating R-factors(gt) etc. and is not relevant to the choice of reflections for refinement. R-factors based on F^2^ are statistically about twice as large as those based on F, and R- factors based on ALL data will be even larger.

### (II) 3-Bromo-N-[2-(trifluoromethyl)phenyl]benzamide Fractional atomic coordinates and isotropic or equivalent isotropic displacement parameters (Å^2^)

**Table:**

	*x*	*y*	*z*	*U*_iso_*/*U*_eq_	
H1	1.167 (2)	0.262 (5)	0.0800 (14)	0.022 (7)*	
Br1	0.85044 (2)	0.78177 (6)	0.240898 (12)	0.02952 (16)	
F3	1.32385 (11)	0.0139 (3)	0.09354 (7)	0.0309 (4)	
F1	1.46295 (12)	0.0991 (4)	0.06107 (8)	0.0414 (4)	
O1	1.10042 (14)	0.8797 (4)	0.07865 (8)	0.0262 (4)	
C12	1.1116 (2)	0.2628 (5)	0.25054 (12)	0.0254 (6)	
H12	1.1500	0.1231	0.2784	0.031*	
F2	1.41294 (13)	0.3932 (3)	0.12181 (7)	0.0379 (4)	
C14	1.3768 (2)	0.2193 (5)	0.07210 (13)	0.0249 (6)	
C13	1.1429 (2)	0.3313 (5)	0.19641 (12)	0.0240 (5)	
H13	1.2016	0.2364	0.1869	0.029*	
C3	1.3518 (2)	0.4176 (6)	−0.03571 (12)	0.0285 (6)	
H3	1.4176	0.3321	−0.0371	0.034*	
N1	1.16903 (15)	0.4434 (4)	0.07018 (9)	0.0210 (4)	
C9	1.0021 (2)	0.6762 (5)	0.17009 (12)	0.0232 (5)	
H9	0.9649	0.8205	0.1431	0.028*	
C8	1.08811 (17)	0.5397 (5)	0.15593 (11)	0.0199 (5)	
C1	1.21188 (18)	0.4948 (5)	0.01743 (11)	0.0207 (5)	
C11	1.0248 (2)	0.3955 (5)	0.26468 (11)	0.0267 (5)	
H11	1.0030	0.3468	0.3016	0.032*	
C6	1.1575 (2)	0.6606 (5)	−0.03303 (12)	0.0254 (5)	
H6	1.0908	0.7434	−0.0328	0.030*	
C10	0.97086 (18)	0.6007 (5)	0.22362 (11)	0.0232 (5)	
C5	1.2015 (2)	0.7035 (6)	−0.08358 (13)	0.0297 (6)	
H5	1.1646	0.8180	−0.1178	0.036*	
C2	1.31040 (18)	0.3751 (5)	0.01629 (11)	0.0217 (5)	
C4	1.2976 (2)	0.5838 (6)	−0.08530 (12)	0.0317 (6)	
H4	1.3264	0.6155	−0.1205	0.038*	
C7	1.11912 (18)	0.6373 (5)	0.09788 (11)	0.0201 (5)	

### (II) 3-Bromo-N-[2-(trifluoromethyl)phenyl]benzamide Atomic displacement parameters (Å^2^)

**Table:**

	*U*^11^	*U*^22^	*U*^33^	*U*^12^	*U*^13^	*U*^23^
Br1	0.0248 (2)	0.0363 (2)	0.0329 (2)	0.00046 (9)	0.01727 (14)	−0.00145 (10)
F3	0.0284 (7)	0.0269 (8)	0.0370 (8)	0.0009 (6)	0.0073 (6)	0.0084 (6)
F1	0.0305 (8)	0.0536 (11)	0.0446 (9)	0.0194 (8)	0.0177 (7)	0.0089 (8)
O1	0.0314 (9)	0.0173 (9)	0.0350 (10)	0.0019 (7)	0.0179 (7)	0.0024 (7)
C12	0.0288 (15)	0.0243 (13)	0.0234 (14)	0.0018 (10)	0.0071 (11)	0.0019 (9)
F2	0.0466 (9)	0.0294 (8)	0.0297 (8)	−0.0022 (7)	−0.0048 (7)	−0.0037 (6)
C14	0.0235 (13)	0.0246 (13)	0.0281 (14)	0.0012 (10)	0.0093 (11)	−0.0042 (9)
C13	0.0218 (12)	0.0228 (12)	0.0286 (13)	−0.0004 (10)	0.0085 (10)	−0.0024 (10)
C3	0.0260 (12)	0.0333 (15)	0.0295 (13)	−0.0006 (11)	0.0134 (10)	−0.0044 (11)
N1	0.0236 (10)	0.0167 (10)	0.0262 (10)	0.0021 (8)	0.0130 (8)	0.0024 (8)
C9	0.0239 (12)	0.0191 (11)	0.0283 (13)	−0.0015 (9)	0.0100 (10)	−0.0013 (9)
C8	0.0180 (11)	0.0183 (11)	0.0250 (12)	−0.0041 (9)	0.0085 (9)	−0.0012 (9)
C1	0.0223 (11)	0.0181 (11)	0.0234 (11)	−0.0035 (9)	0.0093 (9)	−0.0035 (9)
C11	0.0305 (13)	0.0291 (14)	0.0207 (12)	−0.0075 (11)	0.0066 (10)	−0.0018 (10)
C6	0.0247 (12)	0.0238 (12)	0.0278 (13)	0.0004 (10)	0.0069 (10)	−0.0005 (10)
C10	0.0204 (11)	0.0241 (13)	0.0281 (12)	−0.0037 (10)	0.0117 (10)	−0.0042 (10)
C5	0.0359 (16)	0.0322 (14)	0.0207 (14)	−0.0005 (11)	0.0066 (11)	0.0026 (10)
C2	0.0211 (11)	0.0214 (12)	0.0235 (12)	−0.0008 (10)	0.0076 (9)	−0.0037 (9)
C4	0.0349 (14)	0.0394 (15)	0.0249 (13)	−0.0041 (12)	0.0151 (11)	−0.0005 (11)
C7	0.0176 (11)	0.0176 (12)	0.0264 (12)	−0.0039 (9)	0.0077 (9)	−0.0016 (9)

### (II) 3-Bromo-N-[2-(trifluoromethyl)phenyl]benzamide Geometric parameters (Å, º)

**Table:**

Br1—C10	1.900 (2)	N1—C1	1.424 (3)
F3—C14	1.342 (3)	N1—H1	0.89 (3)
F1—C14	1.328 (3)	C9—C10	1.381 (3)
O1—C7	1.226 (3)	C9—C8	1.390 (3)
C12—C13	1.386 (4)	C9—H9	0.9500
C12—C11	1.389 (4)	C8—C7	1.500 (3)
C12—H12	0.9500	C1—C6	1.392 (3)
F2—C14	1.350 (3)	C1—C2	1.402 (3)
C14—C2	1.497 (4)	C11—C10	1.386 (4)
C13—C8	1.394 (3)	C11—H11	0.9500
C13—H13	0.9500	C6—C5	1.383 (4)
C3—C4	1.380 (4)	C6—H6	0.9500
C3—C2	1.392 (3)	C5—C4	1.376 (4)
C3—H3	0.9500	C5—H5	0.9500
N1—C7	1.353 (3)	C4—H4	0.9500
			
C13—C12—C11	121.0 (2)	C6—C1—C2	119.5 (2)
C13—C12—H12	119.5	C6—C1—N1	121.2 (2)
C11—C12—H12	119.5	C2—C1—N1	119.3 (2)
F1—C14—F3	106.3 (2)	C10—C11—C12	118.4 (2)
F1—C14—F2	105.9 (2)	C10—C11—H11	120.8
F3—C14—F2	105.3 (2)	C12—C11—H11	120.8
F1—C14—C2	113.4 (2)	C5—C6—C1	119.4 (2)
F3—C14—C2	113.9 (2)	C5—C6—H6	120.3
F2—C14—C2	111.5 (2)	C1—C6—H6	120.3
C12—C13—C8	119.8 (2)	C9—C10—C11	121.6 (2)
C12—C13—H13	120.1	C9—C10—Br1	118.99 (19)
C8—C13—H13	120.1	C11—C10—Br1	119.40 (18)
C4—C3—C2	120.1 (2)	C4—C5—C6	121.4 (2)
C4—C3—H3	119.9	C4—C5—H5	119.3
C2—C3—H3	119.9	C6—C5—H5	119.3
C7—N1—C1	125.2 (2)	C3—C2—C1	119.9 (2)
C7—N1—H1	120 (2)	C3—C2—C14	118.6 (2)
C1—N1—H1	114 (2)	C1—C2—C14	121.4 (2)
C10—C9—C8	119.6 (2)	C5—C4—C3	119.7 (2)
C10—C9—H9	120.2	C5—C4—H4	120.1
C8—C9—H9	120.2	C3—C4—H4	120.1
C9—C8—C13	119.6 (2)	O1—C7—N1	123.9 (2)
C9—C8—C7	116.6 (2)	O1—C7—C8	120.5 (2)
C13—C8—C7	123.7 (2)	N1—C7—C8	115.6 (2)
			
C11—C12—C13—C8	1.2 (4)	N1—C1—C2—C3	178.3 (2)
C10—C9—C8—C13	−1.0 (3)	C6—C1—C2—C14	173.9 (2)
C10—C9—C8—C7	−178.6 (2)	N1—C1—C2—C14	−6.4 (3)
C12—C13—C8—C9	−0.3 (4)	F1—C14—C2—C3	−10.1 (3)
C12—C13—C8—C7	177.1 (2)	F3—C14—C2—C3	−131.8 (2)
C7—N1—C1—C6	−40.8 (3)	F2—C14—C2—C3	109.3 (3)
C7—N1—C1—C2	139.5 (2)	F1—C14—C2—C1	174.5 (2)
C13—C12—C11—C10	−0.7 (4)	F3—C14—C2—C1	52.8 (3)
C2—C1—C6—C5	0.2 (4)	F2—C14—C2—C1	−66.1 (3)
N1—C1—C6—C5	−179.5 (2)	C6—C5—C4—C3	−0.1 (4)
C8—C9—C10—C11	1.5 (4)	C2—C3—C4—C5	−1.1 (4)
C8—C9—C10—Br1	−178.14 (17)	C1—N1—C7—O1	3.6 (4)
C12—C11—C10—C9	−0.6 (4)	C1—N1—C7—C8	−175.5 (2)
C12—C11—C10—Br1	178.98 (18)	C9—C8—C7—O1	27.0 (3)
C1—C6—C5—C4	0.5 (4)	C13—C8—C7—O1	−150.5 (2)
C4—C3—C2—C1	1.8 (4)	C9—C8—C7—N1	−153.9 (2)
C4—C3—C2—C14	−173.6 (2)	C13—C8—C7—N1	28.5 (3)
C6—C1—C2—C3	−1.4 (3)		

### (II) 3-Bromo-N-[2-(trifluoromethyl)phenyl]benzamide Hydrogen-bond geometry (Å, º)

**Table:**

*D*—H···*A*	*D*—H	H···*A*	*D*···*A*	*D*—H···*A*
N1—H1···O1^i^	0.89 (2)	2.00 (2)	2.835 (2)	156 (3)

Symmetry code: (i) *x*, *y*−1, *z*.

### (III) 3-Iodo-N-[2-(trifluoromethyl)phenyl]benzamide Crystal data

**Table:**

C_14_H_9_F_3_INO	Prism
*M**_r_* = 391.12	*D*_x_ = 1.908 Mg m^−^^3^
Monoclinic, *P*2_1_/*n*	Melting point: 393 K
*a* = 13.3358 (6) Å	Cu *K*α radiation, λ = 1.54178 Å
*b* = 4.7471 (2) Å	Cell parameters from 131 reflections
*c* = 22.3558 (10) Å	θ = 6.2–64.3°
β = 105.848 (2)°	µ = 18.78 mm^−^^1^
*V* = 1361.47 (10) Å^3^	*T* = 173 K
*Z* = 4	Prism, colourless
*F*(000) = 752	0.27 × 0.22 × 0.18 mm

### (III) 3-Iodo-N-[2-(trifluoromethyl)phenyl]benzamide Data collection

**Table:**

Bruker APEXII CCD diffractometer	2223 independent reflections
Radiation source: fine-focus sealed tube	2124 reflections with *I* > 2σ(*I*)
Graphite monochromator	*R*_int_ = 0.053
phi and φ scans	θ_max_ = 64.3°, θ_min_ = 6.2°
Absorption correction: multi-scan (SADABS; Bruker, 2009)	*h* = −15→14
*T*_min_ = 0.081, *T*_max_ = 0.133	*k* = −5→5
7120 measured reflections	*l* = −25→25

### (III) 3-Iodo-N-[2-(trifluoromethyl)phenyl]benzamide Refinement

**Table:**

Refinement on *F*^2^	Primary atom site location: structure-invariant direct methods
Least-squares matrix: full	Secondary atom site location: difference Fourier map
*R*[*F*^2^ > 2σ(*F*^2^)] = 0.043	Hydrogen site location: inferred from neighbouring sites
*wR*(*F*^2^) = 0.109	H atoms treated by a mixture of independent and constrained refinement
*S* = 1.09	*w* = 1/[σ^2^(*F*_o_^2^) + (0.0788*P*)^2^] where *P* = (*F*_o_^2^ + 2*F*_c_^2^)/3
2223 reflections	(Δ/σ)_max_ < 0.001
185 parameters	Δρ_max_ = 1.84 e Å^−^^3^
1 restraint	Δρ_min_ = −1.41 e Å^−^^3^

### (III) 3-Iodo-N-[2-(trifluoromethyl)phenyl]benzamide Special details

**Table:**

Geometry. All s.u.'s (except the s.u. in the dihedral angle between two l.s. planes) are estimated using the full covariance matrix. The cell s.u.'s are taken into account individually in the estimation of s.u.'s in distances, angles and torsion angles; correlations between s.u.'s in cell parameters are only used when they are defined by crystal symmetry. An approximate (isotropic) treatment of cell s.u.'s is used for estimating s.u.'s involving l.s. planes.
Refinement. Refinement of F^2^ against ALL reflections. The weighted R-factor wR and goodness of fit S are based on F^2^, conventional R-factors R are based on F, with F set to zero for negative F^2^. The threshold expression of F^2^ > 2σ(F^2^) is used only for calculating R-factors(gt) etc. and is not relevant to the choice of reflections for refinement. R-factors based on F^2^ are statistically about twice as large as those based on F, and R- factors based on ALL data will be even larger.

### (III) 3-Iodo-N-[2-(trifluoromethyl)phenyl]benzamide Fractional atomic coordinates and isotropic or equivalent isotropic displacement parameters (Å^2^)

**Table:**

	*x*	*y*	*z*	*U*_iso_*/*U*_eq_	
C12	1.1208 (4)	0.7461 (9)	0.2483 (2)	0.0187 (10)	
H12	1.1593	0.8842	0.2760	0.022*	
H1	1.167 (4)	0.751 (7)	0.081 (2)	0.017 (13)*	
I1	0.85495 (2)	0.21117 (7)	0.240621 (11)	0.02136 (18)	
F1	1.32578 (18)	0.9967 (6)	0.09091 (11)	0.0280 (6)	
F3	1.4613 (2)	0.8794 (9)	0.06351 (14)	0.0448 (8)	
C13	1.1487 (4)	0.6826 (9)	0.19429 (19)	0.0196 (9)	
H13	1.2047	0.7794	0.1847	0.024*	
O1	1.1058 (2)	0.1341 (7)	0.07951 (13)	0.0225 (7)	
C3	1.3480 (3)	0.5671 (12)	−0.03282 (19)	0.0270 (11)	
H3	1.4136	0.6425	−0.0335	0.032*	
F2	1.4039 (2)	0.6087 (7)	0.12348 (11)	0.0360 (6)	
C9	1.0116 (3)	0.3381 (9)	0.16847 (18)	0.0177 (8)	
H9	0.9745	0.1950	0.1417	0.021*	
C8	1.0943 (3)	0.4774 (9)	0.15463 (16)	0.0147 (8)	
C14	1.3742 (4)	0.7780 (9)	0.0729 (2)	0.0202 (10)	
N1	1.1697 (2)	0.5723 (8)	0.06981 (14)	0.0165 (7)	
C11	1.0390 (3)	0.6134 (10)	0.26236 (17)	0.0204 (9)	
H11	1.0203	0.6594	0.2992	0.025*	
C6	1.1542 (3)	0.3508 (10)	−0.03154 (19)	0.0216 (9)	
H6	1.0877	0.2776	−0.0320	0.026*	
C7	1.1231 (3)	0.3789 (10)	0.09730 (18)	0.0165 (9)	
C10	0.9841 (3)	0.4115 (9)	0.22197 (17)	0.0167 (8)	
C1	1.2104 (3)	0.5142 (9)	0.01811 (16)	0.0154 (8)	
C5	1.1976 (5)	0.2967 (10)	−0.0806 (2)	0.0277 (11)	
H5	1.1602	0.1826	−0.1143	0.033*	
C2	1.3078 (3)	0.6222 (10)	0.01769 (17)	0.0184 (8)	
C4	1.2923 (4)	0.4035 (12)	−0.08166 (18)	0.0302 (11)	
H4	1.3197	0.3651	−0.1158	0.036*	

### (III) 3-Iodo-N-[2-(trifluoromethyl)phenyl]benzamide Atomic displacement parameters (Å^2^)

**Table:**

	*U*^11^	*U*^22^	*U*^33^	*U*^12^	*U*^13^	*U*^23^
C12	0.016 (2)	0.022 (3)	0.017 (2)	0.0021 (16)	0.0033 (19)	−0.0030 (16)
I1	0.0206 (2)	0.0284 (3)	0.0216 (2)	0.00032 (9)	0.01687 (16)	0.00060 (9)
F1	0.0282 (13)	0.0237 (15)	0.0317 (12)	−0.0011 (11)	0.0074 (11)	−0.0056 (11)
F3	0.0302 (15)	0.067 (2)	0.0431 (16)	−0.0210 (16)	0.0205 (13)	−0.0115 (17)
C13	0.027 (2)	0.018 (3)	0.017 (2)	0.0013 (18)	0.0107 (18)	0.0035 (16)
O1	0.0341 (17)	0.0156 (17)	0.0265 (15)	−0.0004 (14)	0.0233 (13)	−0.0033 (13)
C3	0.025 (2)	0.044 (3)	0.0185 (19)	0.008 (2)	0.0166 (17)	0.006 (2)
F2	0.0484 (16)	0.0305 (17)	0.0216 (12)	0.0002 (13)	−0.0034 (11)	0.0045 (12)
C9	0.019 (2)	0.019 (2)	0.0179 (19)	0.0030 (17)	0.0096 (16)	0.0005 (16)
C8	0.0173 (17)	0.016 (2)	0.0145 (16)	0.0041 (16)	0.0104 (14)	0.0004 (15)
C14	0.020 (3)	0.026 (3)	0.017 (2)	−0.0005 (17)	0.0094 (19)	0.0018 (16)
N1	0.0217 (16)	0.017 (2)	0.0167 (15)	−0.0002 (14)	0.0150 (13)	−0.0016 (14)
C11	0.022 (2)	0.028 (3)	0.0143 (18)	0.0093 (19)	0.0097 (16)	−0.0019 (18)
C6	0.024 (2)	0.024 (3)	0.0190 (19)	0.0005 (19)	0.0092 (17)	−0.0009 (18)
C7	0.0177 (19)	0.017 (2)	0.0181 (19)	0.0047 (18)	0.0108 (15)	0.0015 (18)
C10	0.0164 (17)	0.020 (2)	0.0187 (18)	0.0016 (17)	0.0133 (15)	0.0041 (17)
C1	0.0194 (18)	0.017 (2)	0.0130 (16)	0.0061 (16)	0.0099 (15)	0.0036 (15)
C5	0.040 (3)	0.033 (3)	0.011 (2)	0.004 (2)	0.009 (2)	−0.0045 (17)
C2	0.024 (2)	0.019 (2)	0.0157 (18)	0.0059 (18)	0.0097 (16)	0.0016 (17)
C4	0.037 (2)	0.044 (3)	0.0166 (19)	0.010 (2)	0.0203 (18)	0.002 (2)

### (III) 3-Iodo-N-[2-(trifluoromethyl)phenyl]benzamide Geometric parameters (Å, º)

**Table:**

C12—H12	0.9500	C9—C10	1.387 (5)
C12—C13	1.390 (6)	C8—C7	1.509 (5)
C12—C11	1.368 (7)	C14—C2	1.502 (6)
I1—C10	2.106 (4)	N1—H1	0.89 (3)
F1—C14	1.342 (5)	N1—C7	1.348 (6)
F3—C14	1.326 (6)	N1—C1	1.431 (4)
C13—H13	0.9500	C11—H11	0.9500
C13—C8	1.382 (6)	C11—C10	1.382 (6)
O1—C7	1.230 (6)	C6—H6	0.9500
C3—H3	0.9500	C6—C1	1.393 (6)
C3—C2	1.400 (5)	C6—C5	1.397 (6)
C3—C4	1.380 (7)	C1—C2	1.399 (6)
F2—C14	1.356 (5)	C5—H5	0.9500
C9—H9	0.9500	C5—C4	1.368 (8)
C9—C8	1.391 (6)	C4—H4	0.9500
			
C13—C12—H12	119.4	C12—C11—H11	120.6
C11—C12—H12	119.4	C12—C11—C10	118.9 (3)
C11—C12—C13	121.2 (5)	C10—C11—H11	120.6
C12—C13—H13	120.3	C1—C6—H6	120.7
C8—C13—C12	119.5 (4)	C1—C6—C5	118.7 (4)
C8—C13—H13	120.3	C5—C6—H6	120.7
C2—C3—H3	119.9	O1—C7—C8	119.9 (4)
C4—C3—H3	119.9	O1—C7—N1	124.4 (3)
C4—C3—C2	120.1 (4)	N1—C7—C8	115.6 (4)
C8—C9—H9	120.5	C9—C10—I1	118.7 (3)
C10—C9—H9	120.5	C11—C10—I1	120.0 (2)
C10—C9—C8	119.0 (4)	C11—C10—C9	121.3 (4)
C13—C8—C9	120.1 (3)	C6—C1—N1	120.7 (3)
C13—C8—C7	123.5 (4)	C6—C1—C2	119.8 (3)
C9—C8—C7	116.3 (4)	C2—C1—N1	119.5 (3)
F1—C14—F2	105.2 (3)	C6—C5—H5	119.1
F1—C14—C2	113.8 (4)	C4—C5—C6	121.9 (4)
F3—C14—F1	106.3 (4)	C4—C5—H5	119.1
F3—C14—F2	106.2 (4)	C3—C2—C14	119.0 (4)
F3—C14—C2	113.2 (4)	C1—C2—C3	119.8 (4)
F2—C14—C2	111.5 (4)	C1—C2—C14	121.0 (3)
C7—N1—H1	117 (3)	C3—C4—H4	120.2
C7—N1—C1	124.1 (4)	C5—C4—C3	119.7 (4)
C1—N1—H1	118 (3)	C5—C4—H4	120.2
			
C12—C13—C8—C9	0.7 (6)	N1—C1—C2—C14	5.1 (6)
C12—C13—C8—C7	−175.6 (4)	C11—C12—C13—C8	−1.4 (7)
C12—C11—C10—I1	−178.2 (3)	C6—C1—C2—C3	0.4 (6)
C12—C11—C10—C9	1.2 (6)	C6—C1—C2—C14	−174.7 (4)
F1—C14—C2—C3	129.8 (4)	C6—C5—C4—C3	0.7 (8)
F1—C14—C2—C1	−55.0 (5)	C7—N1—C1—C6	43.4 (6)
F3—C14—C2—C3	8.3 (6)	C7—N1—C1—C2	−136.4 (4)
F3—C14—C2—C1	−176.5 (4)	C10—C9—C8—C13	0.9 (6)
C13—C12—C11—C10	0.5 (7)	C10—C9—C8—C7	177.4 (4)
C13—C8—C7—O1	148.7 (4)	C1—N1—C7—O1	−3.5 (6)
C13—C8—C7—N1	−29.7 (6)	C1—N1—C7—C8	174.8 (3)
F2—C14—C2—C3	−111.3 (5)	C1—C6—C5—C4	−1.1 (7)
F2—C14—C2—C1	63.9 (5)	C5—C6—C1—N1	−179.2 (4)
C9—C8—C7—O1	−27.7 (5)	C5—C6—C1—C2	0.6 (6)
C9—C8—C7—N1	153.9 (4)	C2—C3—C4—C5	0.4 (8)
C8—C9—C10—I1	177.5 (3)	C4—C3—C2—C14	174.4 (4)
C8—C9—C10—C11	−1.9 (6)	C4—C3—C2—C1	−0.9 (7)
N1—C1—C2—C3	−179.8 (4)		

### (III) 3-Iodo-N-[2-(trifluoromethyl)phenyl]benzamide Hydrogen-bond geometry (Å, º)

**Table:**

*D*—H···*A*	*D*—H	H···*A*	*D*···*A*	*D*—H···*A*
N1—H1···O1^i^	0.89 (3)	1.99 (4)	2.826 (5)	156 (5)

Symmetry code: (i) *x*, *y*+1, *z*.
